# Degenerative processes in bioprosthetic mitral valves in juvenile pigs

**DOI:** 10.1186/1749-8090-6-72

**Published:** 2011-05-15

**Authors:** Jesper L Honge, Jonas A Funder, Torben B Pedersen, Mads B Kronborg, J Michael Hasenkam

**Affiliations:** 1 Department of Cardiothoracic and Vascular Surgery, Aarhus University Hospital, Skejby, Denmark; 2 Institute of Clinical Medicin, Aarhus University Hospital, Skejby, Denmark; 3 Department of Cardiology, Aarhus University Hospital, Skejby, Denmark

**Keywords:** Mitral valve, glutaraldehyde, porcine model, calcification

## Abstract

**Background:**

Glutaraldehyde-treated bioprosthetic heart valves are commonly used for replacement of diseased heart valves. However, calcification and wear limit their durability, and the development of new and improved bioprosthetic valve designs is needed and must be evaluated in a reliable animal model. We studied glutaraldehyde-treated valves 6 months after implantation to evaluate bioprosthetic valve complications in the mitral position in juvenile pigs.

**Materials:**

The study material comprised eight, 5-month old, 60-kg pigs. All pigs received a size 27, glutaraldehyde-treated, stented, Carpentier-Edwards S.A.V. mitral valve prosthesis. After six months, echocardiography was performed, and the valves explanted for gross examination, high resolution X-ray, and histological evaluation.

**Results:**

Five pigs survived the follow-up period. Preexplant echocardiography revealed a median peak and mean velocity of 1.61 m/s (range: 1.17-2.00) and 1.20 (SD = ±0.25), respectively, and a median peak and mean pressure difference of 10.42 mmHg (range: 5.83-16.55) and 6.51 mmHg (SD = ±2.57), respectively. Gross examination showed minor thrombotic depositions at two commissures in two valves and at all three commissures in three valves. High resolution X-ray imaging revealed different degrees of calcification in all explanted valves, primarily in the commissural and belly areas. In all valves, histological evaluation demonstrated various degrees of fibrous sheath formation, limited immunological infiltration, and no overgrowth of host endothelium.

**Conclusions:**

Bioprosthetic glutaraldehyde-treated mitral valves can be implanted into the mitral position in pigs and function after 6 months. Echocardiographic data, calcification, and histological examinations were comparable to results obtained in sheep models and human demonstrating the suitability of the porcine model.

## Introduction

Glutaraldehyde-treated bioprosthetic heart valves are commonly used for replacement of diseased heart valves. In the mitral position, glutaraldehyde-treated valves are preferred in elderly patients (>65 years), in patients in whom successful repair is unlikely, in rheumatic disease and endocarditis, and when the use of anticoagulation therapy is contraindicated [1]. The failure rate of current bioprosthetic mitral valves is much higher than that of aortic valves and reoperation is needed in 50% after 15 years [2]. Therefore, the development of new and improved bioprosthetic valve designs is needed. To evaluate bioprosthetic heart valves before clinical implementation, animal testing is preferred. Several species (sheep, dog, and pig) have been chosen for valve evaluation, the sheep model being the most common [3-6]. The sheep model, however, has several limitations. It failed to demonstrate the thrombogenecity of the Medtronic Parallel mechanical valve, which, after implantation in patients, was associated with a high incidence of valve-related thrombosis. It also failed to show a strong inflammatory response toward a decellularized biological valve, Synergraft, which was seen after implantation in children [7,8]. The anatomical and physiological similarities between pigs and humans in terms of heart size, cardiac output, blood pressure, and, in particular, platelet adhesive properties make this model suitable for heart valve evaluation [9-11].

To evaluate new bioprosthetic valves, it is essential to know how these perform in an animal model compared with standard glutaraldehyde-treated valves, which are considered the gold standard of bioprosthetic valves. We, therefore, chose the pig model to thoroughly evaluate standard glutaraldehyde-treated bioprosthetic stented porcine valves in the mitral position in a long-term model.

## Materials and methods

This observational study was conducted in female Danish Landrace/Yorkshire pigs. The study material comprised eight, 5-month old, 60-kg pigs. All pigs received a size 27, glutaraldehyde-treated, stented, Carpentier-Edwards S.A.V. mitral valve prosthesis.

All animal experiments were conducted according to the guidelines given by the Danish Inspectorate for Animal Experimentation and after specific approval from this institution. Qualified animal caretaker personnel monitored the health status of the animals daily during the study period. Analgesics were administered if animals exhibited any sign of pain. In the case of refractory pain or failure to thrive, the animals were euthanized. At the end of the study, the animals were euthanized under anaesthesia.

### Mitral valve implantation

The operative technique and anesthetic treatment have been described elsewhere [5]. In brief, after sedation, intubation and median sternotomy, a cardiopulmonary bypass was performed and the heart arrested with cold crystalloid cardioplegia. A size 27 Carpentier-Edwards mitral bioprosthesis was then implanted through a left atriotomy. Next, the atrium was closed and a DC countershock was given. After approximately 45 minutes of reperfusion, the animal was weaned from the cardiopulmonary bypass and the chest closed in a standard manner. The chest drains were removed when satisfactory hemodynamics were obtained and drain production was below 50 ml/h. The animals were awakened after 4 hours and transported to the farm in the evening. Importantly, no antithrombotic therapy was given during the follow-up period.

### Preexplant analysis

Echocardiography was performed using a commercially available system (Vivid E9, General Electric, Horten, Norway) in supine pigs under anesthesia. Parasternal echocardiograms were obtained by a transthoracic approach, and apical echocardiograms were obtained through a minimal abdominal incision. The study protocol included 2-dimensional echocardiograms with color Doppler images (apical four chamber, two chamber, long axis, as well as parasternal long axis) to evaluate left ventricular ejection fraction and mitral regurgitation using visual assessment by an experienced observer [12]. To measure blood velocities and pressure differences over the artificial valves, continuous-wave Doppler echocardiograms through the center of the mitral ostium were obtained [13]. All echocardiograms were recorded twice including three consecutive heart beats, and analyzed offline using commercial software (Echopac, General Electric-Vingmed, Horten, Norway).

### Explantation

The animals were euthanized after 6 months, and the valves were explanted after administration of an intravenous dose of 10.000 IU unfractionated heparin. Animals that died during the first postoperative week were excluded from the study. If the animals exhibited failure to thrive later than 1 week postoperatively, they were euthanized and the valve was explanted.

### Postexplant analysis

#### Gross Examination

All valves were inspected in situ and photographed after removal. Inspection included gross assessment of fenestration, thrombotic material, and vegetations. The amount of thrombotic material was quantified in terms of size, appearance, and location.

#### Radiography

High Resolution X-ray was performed to evaluate locations and distribution of calcifications using an Xpert 40™, Kubtec technologies, Milford, CT, USA.

#### Histological evaluation

Cusps and housing site tissues were removed from the stent and transected radially. Next, the tissue was fixated in formaldehyde and embedded in paraffin. Hematoxylin-eosin (H&E), vimentin (monoclonal mouse antivimentin), elastin trichrome, von Willebrand factor (polyclonal rabbit antihuman, DAKO) (endothelial cell), smooth muscle cell actin (monoclonal mouse antihuman, DAKO) (smooth muscle cell), and von Kossa stains were used to evaluate areas of recellularization, cell type, structural changes in the trilaminar cusp architecture, and both intrinsic and extrinsic calcification foci. Cells with a clear reduction in basophilia and increase in eosinophilia in H&E stains as well as a negative vimentin stain were considered donor cells. Vimentin-positive cells were therefore considered to be host cells and were afterwards correlated with cell-specific stains to verify their phenotype in order to differentiate between host and donor cells. The specimens were evaluated as a single observer, nonblinded assessment. The equipment used for histological assessment and image capturing was an Olympus BX50 microscope with Olympus Power View II camera.

#### Statistical analysis

Standard descriptive statistics (means, standard deviations, medians, and ranges) were used to characterize the investigated valves. Blood velocity was expressed as median peak and mean velocity. Pressure difference was expressed as median peak and mean pressure difference.

## Results

Eight Carpentier-Edwards S.A.V. mitral valves were implanted into the mitral position. One pig was euthanized after 5 days because of failure to thrive and respiratory insufficiency. The valve was found to be competent without alterations compared with the same valve at the time of implantation. One pig was euthanized after 3 months because of sudden unexplained lower limb paralysis. Gross anatomy and histologic evaluation of the valve from this pig revealed a well-functioning valve prosthesis with a minor thrombosis in the commissural area and no endocarditis. One pig died suddenly after 4 months. Autopsy revealed severe thrombosis of the valve and histological evaluation showed infective endocarditis and calcification of all cusps. Five pigs survived the 6-month follow-up.

### Echocardiography

The median peak and mean velocities over the valves were 1.61 m/s (range: 1.17-2.00) and 1.20 (SD = ±0.25), respectively, and the median peak and mean pressure differences over the valves were 10.42 mmHg (range: 5.83-16.55) and 6.51 mmHg (SD = ±2.57), respectively. All velocities and pressure differences were within the normal ranges for Carpentier-Edwards mitral valves implanted into humans (14). Three animals had mild central mitral regurgitation, and all animals had normal left ventricular ejection fractions. No paravalvular leaks were observed.

### Gross pathology

**Figure 1:**
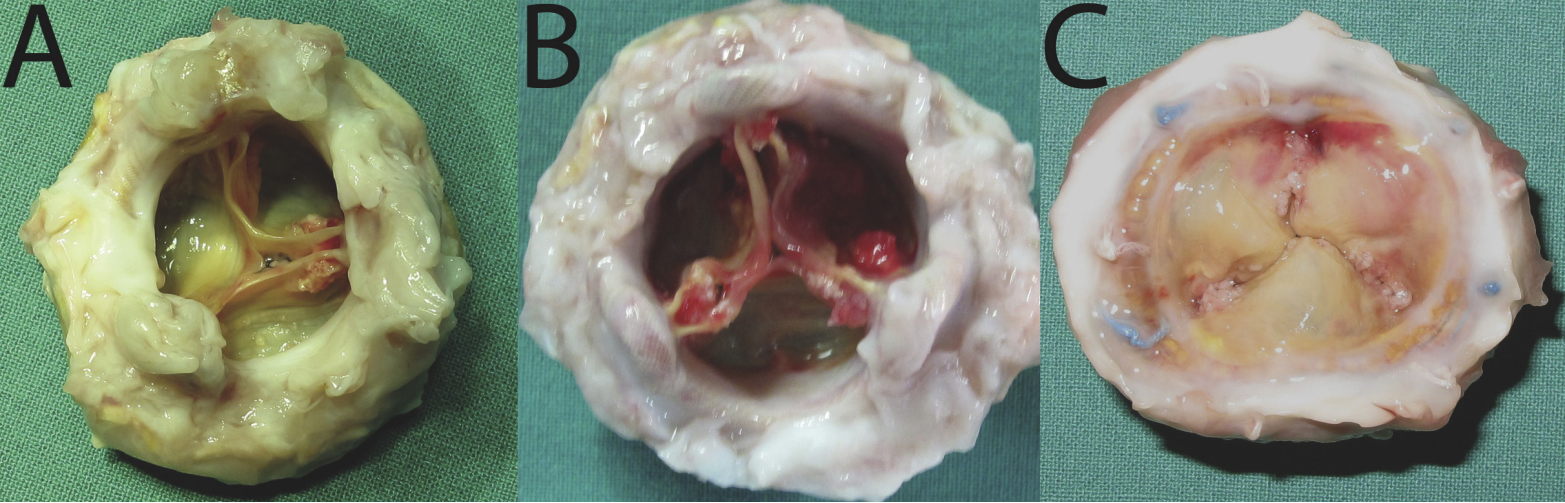
**Gross anatomy 6 months after implantation**. A) Minor thrombotic deposits with calcifications are seen at two of the commissures. B) Severe thrombocalcific deposits involving the belly area can be observed. C) A triradiate pattern of thrombocalcific deposits on the inflow aspect of the coaptation area.

All valves were without fenestrations or tears. Minor thrombotic depositions were observed at two commissures in two valves and at all three commissures in three valves (Figure 1A &1B). In two of these three valves, the depositions were severe and stretched from the commissures into the cusps, exhibiting a triradiate pattern of thrombus deposition on the inflow aspect at the belly and coaptation areas of the valve (Figure 1C). Four valves had minor fibrin depositions in the belly area of one or more of the cusps. Hemorrhages were seen in one cusp in three valves and two cusps in one valve. The stent posts and sewing ring were in all cases covered with a layer of fibrous sheath stretching from the endocardium toward the cusps.

### Radiography

**Figure 2:**
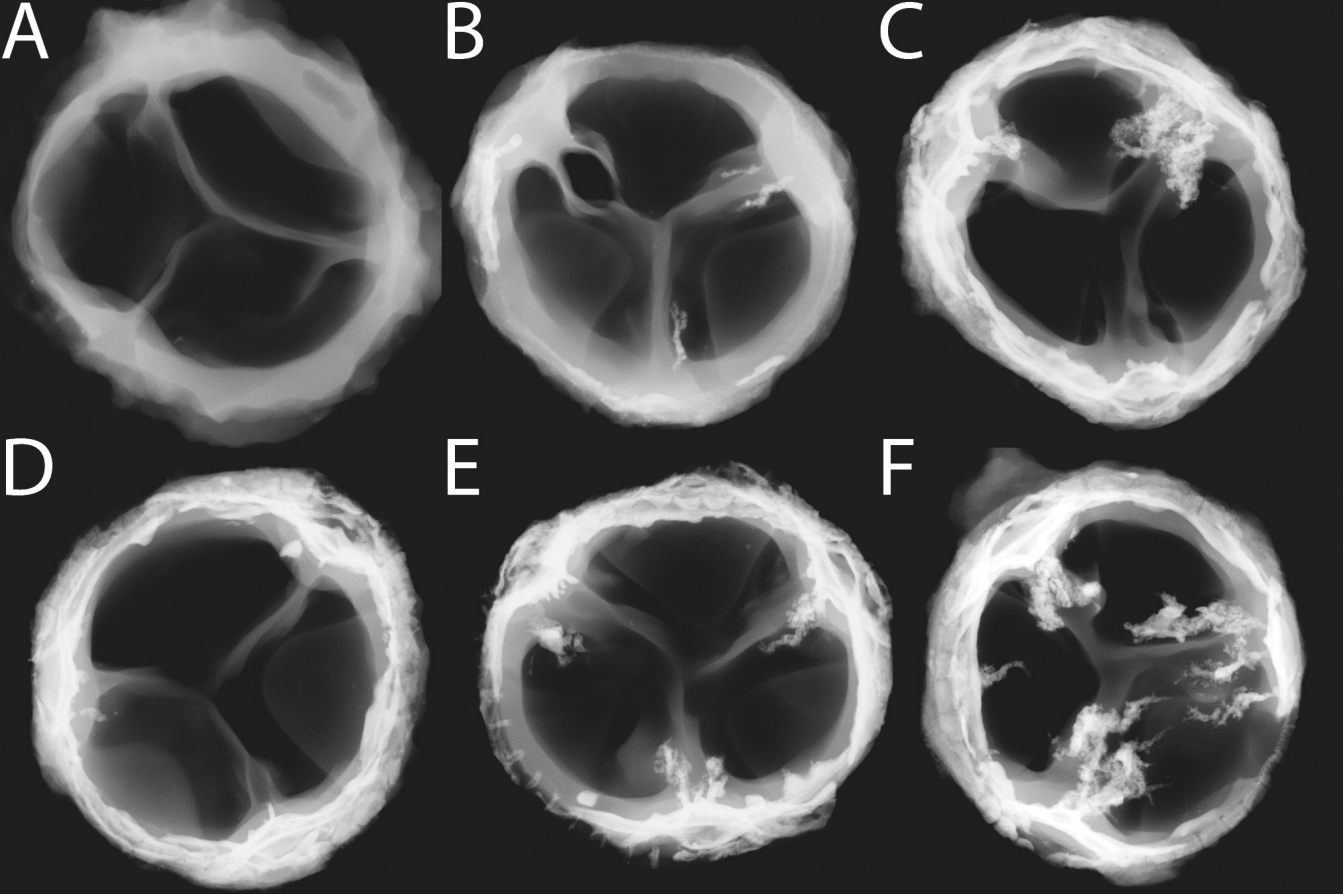
**High resolution X-ray after the sewing ring and stent have been removed**. A) Not implanted control valve without any calcification. B-D) Minor calcific depositions can be seen at two of the commissures. E-F) Various degrees of calcification involving all three commissures and parts of the belly area.

High resolution X-ray imaging revealed the presence of calcification in variable degrees in all explanted valves compared with high resolution X-ray imaging of a control valve that had not been implanted (Figure 2A). In all valves, calcification was observed in varies degrees in the stent adjacent area. In three valves, minor calcific depositions were seen at two of the commissures (Figure 2B-D). In the other two valves, more severe calcification could be observed at all three commissures, and in both of these, parts of the belly region of either one or all three cusps exhibited calcific depositions (Figure 2E-F).

### Histological evaluation

**Figure 3:**
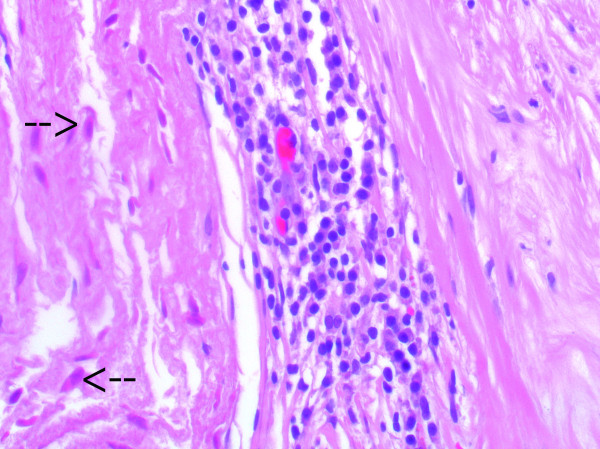
**Histological imaging of the donor tissue (left) and host tissue in the stent-adjacent area (right)**. Non-vital, less basophilic cell remnants can be clearly seen (arrows). Inflammatory cell response is seen between the donor tissue and the fibrous sheath (H&E stain; ×400).

**Figure 4:**
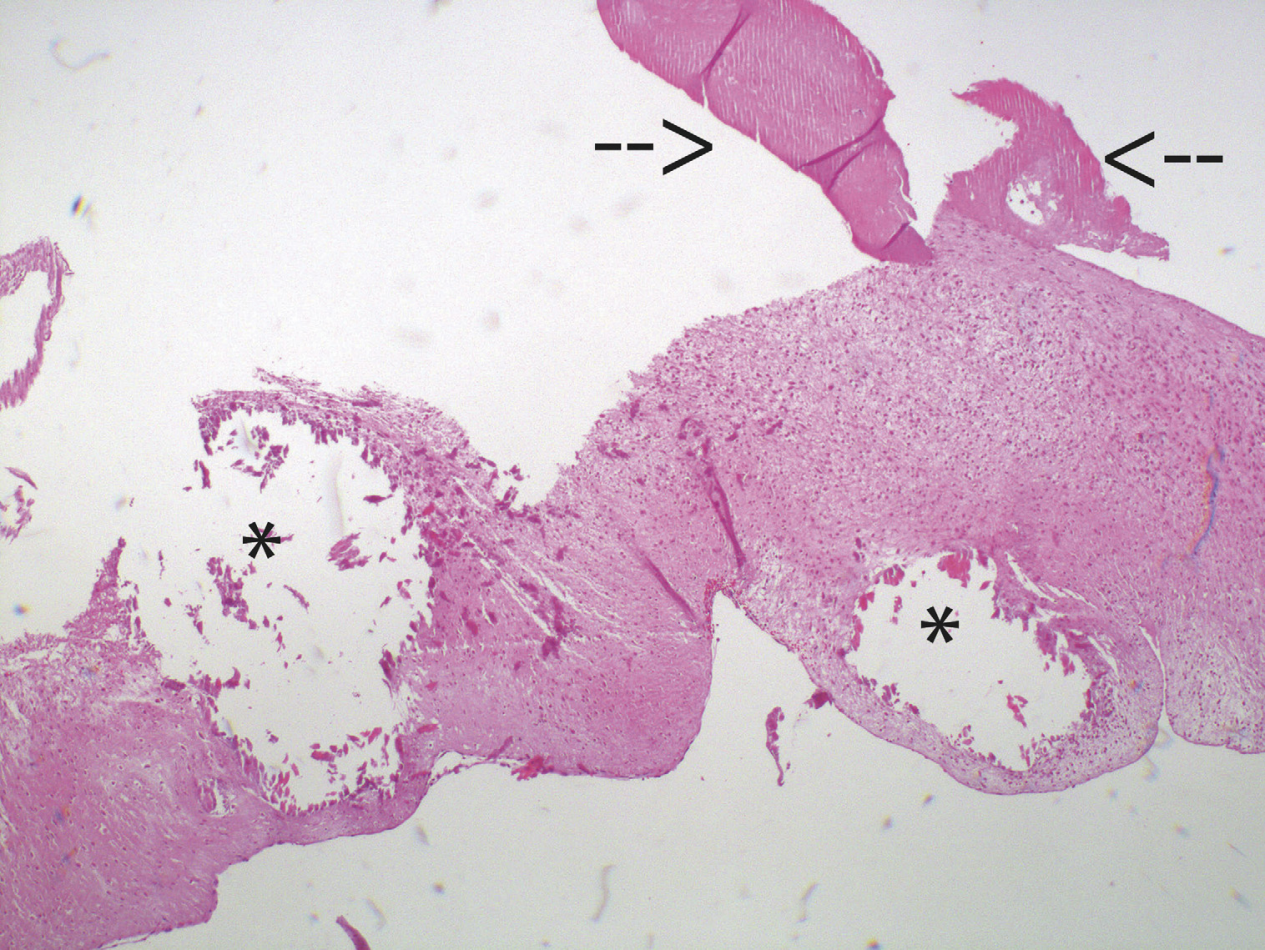
Intrinsic calcification (asterisks) and thrombotic deposits (arrows) of the valve cusp (H&E stain; ×40)

**Figure 5:**
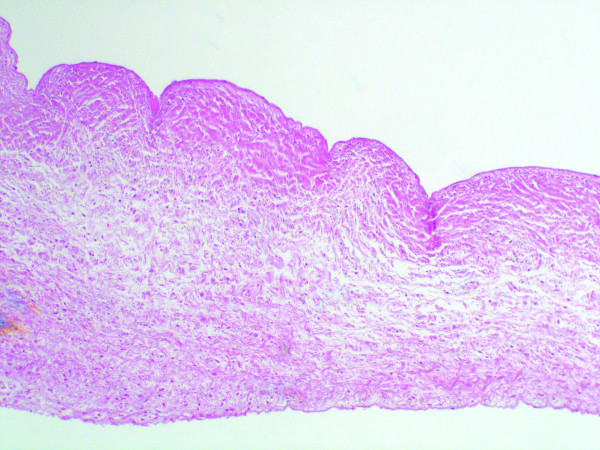
**Histological image of the valve cusp**. The tissue appears well preserved, with corrugations and lack of inflammatory response (H&E stain; ×100)

**Figure 6:**
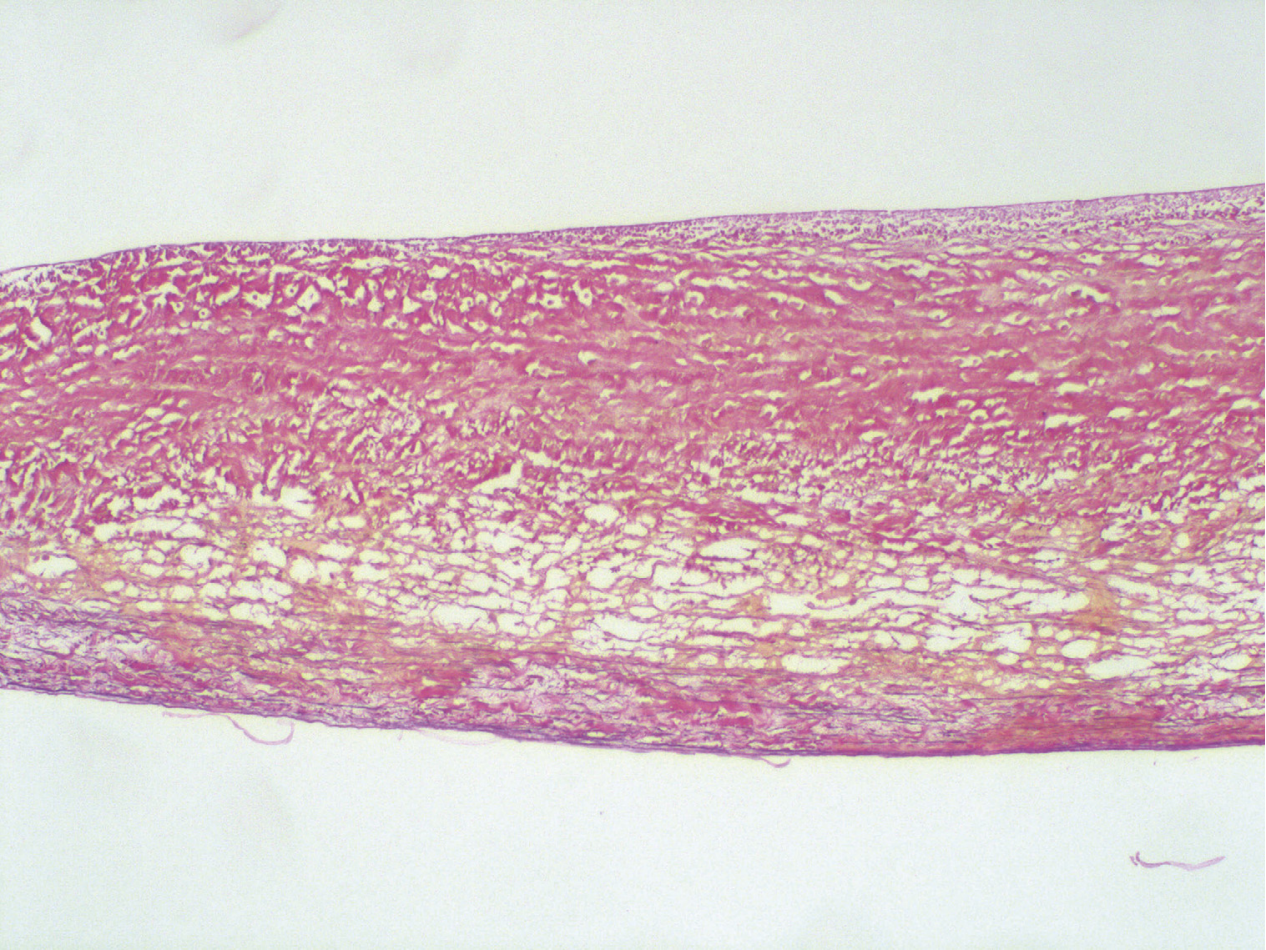
In two valves, fragmentation of the lamina spongiosa is seen, with the absence of corrugations (Weigers stain; ×100)

H&E stain revealed the presence of nonvital donor cell remnants throughout the valve tissue. These cells were characterized by a negative vimentin stain and appeared less basophilic compared with host cells. Fibrous sheath formation was observed in all valves to various degrees; in two valves, the fibrous sheath stretched from both the atrial and ventricular sides to the base of the cusp. Between the donor tissue and the fibrous sheath, inflammatory cells could be seen in small numbers in all valves (Figure 3). Fibroblast ingrowth was limited to the fibrous sheath and as a part of an inflammatory response in the stent-adjacent area. No cusp ingrowth of fibroblasts was seen in any of valves. Host cells were almost absent in the cusp tissue except for in one valve where infiltration of macrophages and lymphocytes could be noted. Only a few minor fibrin depositions were observed on the surface of the valves, except for in one valve where larger depositions could be seen. Von Kossa stains revealed severe calcification of the stent adjacent area, annulus, and myocytes in three valves. Intrinsic calcification of the cusp was observed in two valves (Figure 4). Inflammatory cells consisting of macrophages and lymphocytes were associated with the calcifications seen in the stent-adjacent areas, but no or only very few inflammatory cells were seen in the presence of cusp calcification. Inflammatory cells were observed in the outer parts of all valves and to a larger degree if a myocardial muscle shelf was present. The cusp tissue, collagen, and elastin appeared well preserved, with clear demarcations of the different laminae except for the calcified areas of the cusps (Figure 5). Intracuspal erythrocytes could be seen in the cusps of three valves but in limited amounts. Limited tissue fragmentation and collagen loosening were only observed in the basal part of two valves (Figure 6). Von Willebrand factor-positive cells could only be seen superimposing parts of the fibrous sheath. No single von Willebrandt factor-positive cells or continuous single-cell layer could be seen in any of the cusps.

### Comment

In this study, we evaluated bioprosthetic glutaraldehyde-treated valves in the mitral position in juvenile pigs for valve complications 6 months after implantation. Few have studied bioprosthetic valves in the mitral position in pigs, and the present study is the first to thoroughly evaluate the performance of glutaraldehyde-treated valves in a long-term porcine model [15-17].

Using high resolution X-ray imaging and von Kossa stain, we found that calcification was apparent in all explanted valves. Calcification of the commissures could be seen in all valves to various degrees and involved the belly area of the cusps in two valves. Thrombotic deposits were especially prominent in connection with the commissural calcifications. Weber et al. found calcification in the aortic area in six of nine Mosaic valves explanted after 20 weeks in a sheep model, and in no cases was cusp calcification observed. The Hancock Standard valve was tested in the same study, and here calcification was focused in the aortic wall and commissures and occasionally at the base of the cusps [18]. Flaming et al. found that calcification were preferentially located at the commissures and present in two of six Perimount valves and four of nine Trilogy valves after implantation in sheep [6]. Additionally, cusp and wall-adjacent calcifications were less frequent. These findings by other groups correspond very well with those of the present study, and this demonstrates the suitability of the porcine model for the evaluation of calcification in bioprosthetic valves.

In a large study in sheep, Flameng et al. found by means of quantitative calcium content analysis significantly lower calcification of the cusps than in the aortic wall portions of the valve [19]. Furthermore, the highest calcium levels were found in the commissures, and the lowest calcium levels were found in the edges of the cusps. Although we did not perform any quantitative calcium content analysis, these results correlate well to the spatial distribution of calcification observed in the present study by means of high resolution X-ray.

Histological evaluation of the investigated valves revealed the presence of non-vital donor cell remnants and very scarce amounts of host cell ingrowth. Fibroblasts were only found in the fibrous sheath or as a part of an inflammatory response toward the outer part of the stent-adjacent area, and endothelial cells could only be identified superimposing the fibrous sheath or as von Willebrand-positive cells in very limited numbers. A host cell inflammatory response was apparent in all valves but limited to the stent-adjacent areas except for one valve in which cusp infiltration was observed. Most of the explanted valves in this study presented with well-preserved trilaminar cusp tissue. Inflammatory cells were infrequently seen in the cusps and mostly limited to the stent-adjacent areas and muscle shelf tissues. The tissue preservation of glutaraldehyde-treated valves was also observed in recent sheep studies in which the cusps appeared well-preserved and contained wavy collagen [6,18]. However, Duarte et al. reported trilaminar structure disruption of the Mosaic valve both with and without alpha amino oleic acid (AOA) treatment [20]. Despite meticulous histological examination, we observed almost no endothelial cells except for those located on the surface of fibrous sheaths, and fibroblast ingrowth was limited to the inflammatory host response seen in the stent-adjacent areas of most of the valves. Therefore, recellularization of glutaraldehyde-treated valves in pigs is extremely limited after an implantation period of 6 months, which corresponds well with human studies of bioprosthetic valve endothealization [21].

We found that both blood velocities and pressure differences across the valve after 6 months were comparable with human studies of the Carpentier-Edwards S.A.V. valve in the mitral position as well as previous sheep studies by Duarte et al. and Irwin et al. [20,22]. Flaming et al. found peak velocites (m/s) of 1.34 (0.89, 1.47) and 1.11 (0.75, 1.54) and mean gradients (mmHg) of 3.6 (3.0, 5.2) and 2.4 (1.7, 5.4) for the Perimount and Trilogy valves, respectively, after 5 months in a sheep model. Although a higher mean pressure difference was found in the present study, most likely caused by the thrombotic deposits in some of the valves, we consider our results to be comparable. Additionally, we observed trivial regurgitation in three valves, a finding which was also noted by Weber et al. in another study [14]. In our study, the thrombotic depositions could have caused some degree of commissural fusion leading to a limitation in cusp movement and minimal valve insufficiency.

Our aim was to evaluate the porcine model for long-term testing of bioprosthetic mitral heart valves. The porcine mitral valve anatomy has already been verified to be very similar to human mitral valve anatomy [23,24]. The present study demonstrates that the porcine model is a reliable animal model for long-term bioprosthetic heart valve evaluation and can be used in the future as a relevant, important, and demanding animal model. The findings in the present study speak for the pig as being an animal model for heart valve evaluation that can provide a satisfactory answer to the question of animal testing that will avoid later tragic clinical incidents such as those seen after preclinical sheep experiments. Thorough evaluation of heart valve bioprostheses is critical before any clinical use. Therefore, we consider the present animal model to be very suitable for preclinical bioprosthetic mitral valve testing to ensure proper patient care. Further validation of the porcine model from other groups to support our findings should be performed to verify the pig as a clinically valid animal model. Especially interesting would be the evaluation of other tissue valves, decellularized valves, and tissue-engineered polymer valves because of the recent focus on this topic.

### Study Limitations

A small number of animals were included in this study. However, because of the comparable results between the animals, we consider the number to be sufficient for the evaluation of the porcine model as a tool for bioprosthetic mitral valve evaluation. No anticoagulation therapy was used during the follow-up period, and this could have resulted in the thrombotic deposits observed. A more aggressive anticoagulation strategy might have limited this problem. No baseline echocardiography was performed postoperatively, and hemodynamic comparison with pre-explantation echocardiography was therefore not possible. The growth potential of the pig could result in a patient-prosthesis mismatch, however, we do not consider this a limiting factor, since all surviving pigs thrived well at euthanization, and none of the three animal deaths before the six months follow-up could be related to mitral stenosis.

## Conclusion

Bioprosthetic glutaraldehyde-treated mitral valves can be implanted into the mitral position in pigs and function after a period of 6 months. Echocardiographic data, calcification, and histological examinations were comparable to results obtained in sheep and humans, and we therefore consider the porcine model an appropriate animal model for bioprosthetic mitral valve testing.

## Competing interests

The authors declare that they have no competing interests.

## Authors' contributions

JMH and JAF were both involved in the conception of the study and the study design as well as drafting and revising the article. TBP contributed to the anesthetic treatment and surgical procedures. MBK contributed to the acquisition of echocardiographic data as well as the data analysis. JLH was involved in all the above mentioned study parts. All authors have approved the manuscript.
